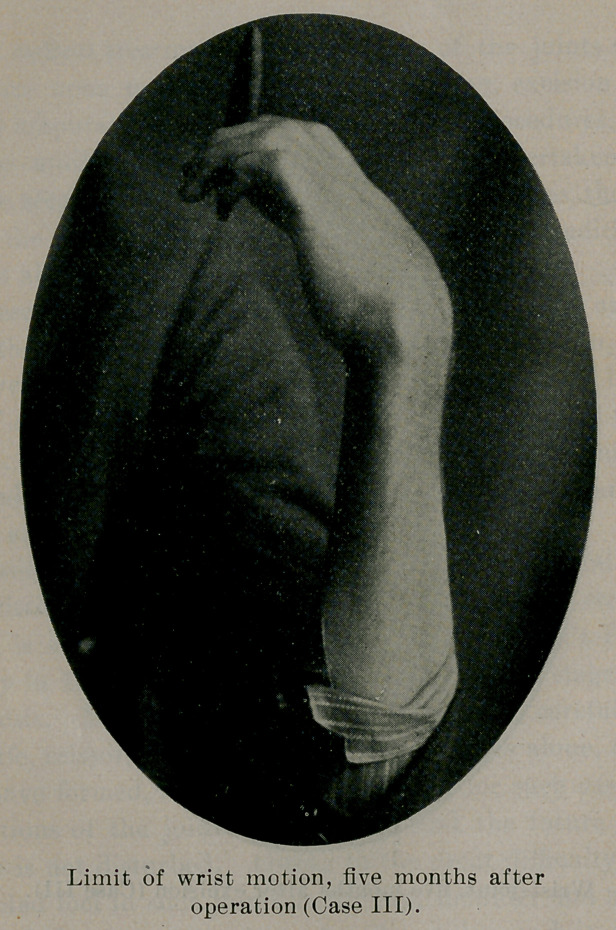# Excision in Tuberculosis of Joints—Hip and Wrist*Read before the Medical Association of Georgia, at Augusta, April, 1901.

**Published:** 1901-06

**Authors:** Howard J. Williams

**Affiliations:** Surgeon to the Macon Hospital, etc., Ex-President to the Medical Association of Georgia, etc., Macon, Ga.


ATLANTA

### Journal-Record of Medicine.

**Successor to Atlanta Medical and Surgical Journal, Established 1855, and Southern Medical Record, Established 1870.**

VOL. III. JUNE, 1901. No. 3.

BERNARD WOLFF, M.D., EDITOR, Nos. 319–20 Prudential.

Published Monthly.

M. B. HUTCHINS, M.D., BUSINESS MANAGER, No. 64 Marietta St.

### *ORIGINAL COMMUNICATIONS.*

### EXCISION IN TUBERCULOSIS OF JOINTS—HIP AND WRIST.*

By HOWARD J. WILLIAMS, A.M., M.D.,

Surgeon to the Macon Hospital, etc., Ex-President to the Medical Association of Georgia, etc., Macon, Ga.

Tuberculosis of the joints, usually secondary to an epiphyseal osteo-tuberculosis, is a peripheral manifestation of the preexistence of the invasion of other organs or tissues by the bacillus tuberculosis. As a direct primary infection, joint or bone tuberculosis is extremely uncommon.

Since this is true, it is also true that the primary focus of tubercle in the internal organs most frequently remains latent, while the greatest danger comes from the bone or joint lesion. Complete destruction of the joint usually resulting, the general system is exposed to septic infection from pus microbes lining old sinuses, important internal organs are subject to amyloid degeneration fol-

*Read before the Medical Association of Georgia, at Augusta, April, 1901.

lowing prolonged joint suppuration, and important organs or tissues are the victims of reinfection by the tubercle bacilli found in old neglected joints. These cases of localized infection are particularly predisposed to the development of acute miliary tuberculosis, and many cases of old neglected joint disease die annually from pulmonary invasion; Billroth says at least 27 per cent. terminate in this way. Adults and those who have an hereditary tendency are more disposed to these sequelæ than children and those who have acquired tuberculosis, though no class is exempt from the dangers. Trauma not infrequently causes relapse or acute dissemination in cases apparently well for years.

Even the radical treatment of diseased joints is not always absolute protection against recurrence of tuberculosis in the lungs or other organs, for the susceptibility to the disease remains after operation. König observed miliary tuberculosis in sixteen cases out of one thousand operations. The writer recalls one case of rapidly fatal tuberculosis of the lungs developing within a few weeks after the removal of a tubercular nodule in bone. Nor are the remote results of operative treatment more encouraging, permanent recovery being rare. Verneuil says: "When we operate on an infected focus, be it osseous, articular, glandular or other tissue, there is great danger from auto-infection from the disturbed local center; the imprisoned microbes being liberated by the traumatism may enter the blood currents and are deposited in various organs;" and further on he says, "patients operated on for tubercular affections never reach old age."

The tubercular process is a multiple invasion metastatic in its course, marked often by periods of latency, attended at times by sudden dissemination, and whose intrinsic tendency is to ultimate death. Such being the character of the disease process, it seems to the writer that the unfortunate sequelæ occasionally observed after operation and the unfavorable results sometimes recorded should not be urged against operative treatment. May not some of the gloomy remote results charged against operative treatment be due to auto-infection from other foci of disease, and is it not possible that some of the cases of early reinfection were the results of imperfect operations? If left to themselves, these neglected joints are still centers of reinfection, while, as Warren says, the successful removal of the local focus will remove the danger of miliary tuberculosis starting from that point. Senn says: "This auto-infection from operative traumatism has been greatly overestimated; if proper precautions are practiced, it is certainly one of the rarest of wound complications." Watson-Cheyne, Tillmans, Park and Warren all claim that local return of the disease shows that the operation has not been thoroughly performed. Furthermore, it should not be forgotten that the removal of a peripheral tubercular lesion has often had a curative effect on similar internal lesions by suppression of reinfection from these foci. Recovery from pulmonary tuberculosis with disappearance of the bacilli from the sputum has been recorded following the radical treatment of joint tuberculosis, and beginning amyloid changes elsewhere have been known to yield to resection of local diseased points.

Later and more exact application of aseptic surgery, greater care now exercised against traumatic infection, and improved methods of operating have greatly reduced the risks of the operative treatment of these local affections. Early and efficient treatment, it seems to the writer, is therefore more urgent than ever in bone and joint tuberculosis. Conservative treatment should yield earlier to absolute wide removal of the infected areas of bone and joint. It is unfortunate that these cases are ever allowed to go on to dislocation, joint suppuration and infected sinuses. These are the cases in which pulmonary infection most frequently develop, and in adults the late complications are more dangerous than the original bone and joint foci. After a brief but fair trial of milder methods of local treatment it is wiser to proceed to methods which are radical in their object and clean in their execution. Even in children, where it is desirable to employ the highest degree of conservatism and removal of bony tissues should be limited to the structures outside of the epiphyseal cartilages so as not to interfere with the future growth of the limb, if there are suspicious centers present beyond this line, the operation should embrace such foci. By early operation fortunately greater conservatism can be exercised; the early removal of a focus in the articular end of a bone need not interfere with the function of the joint, nor will it result in the arrest of the development of the limb. The writer recalls a case in which, eleven years ago, he excised the middle cuneiform and the immediate synovial tissues for local tubercle in a child two years of age; the child is now thirteen years of age and has a perfectly useful foot.

In the radical treatment of tuberculosis of the joints three procedures are open to the surgeon: arthrectomy, excision or resection, and amputation. Unfortunately, these procedures, being regarded as mutilating operations, are usually undertaken after the joints are secondarily infected by suppurating sinuses, the articular ends of the bone broken down, and the general condition of the patient is almost beyond relief.

Of these operations amputation is the most radical, and though it gives the most efficient protection against reinfection, it should be reserved for the graver cases, those which have gone beyond the reach of arthrectomy and excision; cases having local tubercular infection, complicated by the presence of active pulmonary tuberculosis, advanced amyloid degeneration of internal organs, or pronounced anemia.

Arthrectomy, the removal of infected tissues, synovial or bony, with as little disturbance of the healthy surrounding structures as possible, is an operation introduced (at the Children's Hospital at Pillsbury in 1887) to replace excision in the early stages of joint tuberculosis. If the operation is confined to the careful removal, with knife, scissors and chisel, of infected tissues alone, and before fistulæ have formed, it is the ideal operation for such cases. By it the functions of the joint are preserved and the future growth of the limb is not disturbed. Owing to the great difficulty of reaching infected foci in such joints as the hip, shoulder and ankle, it is not regarded as an operation of wide utility, and is at present limited almost exclusively to the knee-joint. Warren objects to arthrectomy in that it is not thorough enough, and that it fails oftener than excision in eradicating the foci of dissemination. Statistics bear him out in this assertion.

Excision, the removal of all infected synovial tissues, together with more or less of the articular ends of bones, is the most universally applicable operation in tubercular joint trouble. If the operation is performed very early, an atypical resection can be performed, only a very thin section of the bone and articular cartilage need be removed, or if the osseous focus can be seen, the chisel or saw may be limited to that portion of the bone containing the nodule.

Wrist-joint, five months after excision (Case III).

These atypical resections or partial excisions give practically the same functional results as arthrectomy. In its performance the knife, scissors, saw and chisel should be used as widely as the needs of the case demand, the curette being only employed to scrape away all the granular and degenerated tissues before the radical steps of the operation are begun. Curetting at this stage of the operation guards the sound tissues about the joint against traumatic dissemination. Cheyne, Warren, Tillmans and Park condemn the curette and kindred instruments in the radical steps of this operation and in arthrectomy. Senn claims that the danger of miliary tuberculosis is greater in all scraping operations. The case of pul-

Limit of wrist motion, five months after operation (Case III).

monary reinfection following operation, recalled by the writer, was the result of curetting.

The great trouble with resection has been that it has usually been delayed too long. Warren says it is unwise to wait for dislocations, joint abscesses and infected sinuses before performing resection, as much of the value of the operation is lost in these late cases. When these complications are present only complete wide excision is called for, regardless of age and the future usefulness of the joint.

If, however, care is taken to preserve the periosteal coverings of the bone and the ligamentous and muscular insertions about these joints uninfected by tubercle bacilli, it is surprising often to see what good functional results may be obtained; the preserved tissues forming good, movable joints and useful limbs, even when typical resections have been performed.

Resection is applicable to the hip, shoulder, elbow and wrist. In the two following cases complete excision of the hip-joint was the only procedure indicated at the time of the operation. In the first case we will see the inevitable local results of temporizing; all radical operative measures were declined at a time when an atypical resection might have saved the joint, preserved the centers of growth and warded off the dangers of possible auto-infection.

CASE 1.—S. B., female, white, of good family history; was first seen by the writer in consultation with Dr. W. F. Holt and Dr. M. A. Clark in the fall of 1898. She was then four years old, and was said to have been in apparently good health up to a few days preceding this visit, when she had some trivial accident. She was taken up, unable to walk, and held the right thigh drawn up to the abdomen, the knee bent, the limb rigidly adducted and rotated inwards; any attempt to straighten the limb caused pain. Chloroform was administered and the head of the femur was found to be dislocated on the dorsum of the ilium.

Oct. 10th, 1898, under chloroform, the dislocation was reduced by manipulation, attended, however, with much difficulty. Extension was applied, the limb being maintained in an abducted position, and the patient confined in bed for one month. A plaster-of-paris casing was then applied and the child allowed to go about on crutches for two months. Apparently there was recovery of function and health.

Early in March, 1899, a swelling was noticed on the outside of the thigh below the greater trochanter, which grew rapidly soft and fluctuating, without pain, heat or discoloration, the child's general health remaining unaffected.

April 7, 1899, this cold abscess was opened, discharging the usual tubercular detritus, its walls thoroughly curetted, scrubbed and irrigated with a sterilized boracic acid wash. The cavity was then injected with a ten per cent. iodoform-glycerine emulsion and sewed up. Primary union followed without any disturbance of function of the joint, and apparent good health continued for the next six months.

Early in January, 1900, the child, then being five and a half years old, began to cry with pain in the knee at night and to limp. A sinus formed in the scar of the incision on the thigh, and the general health began to break down.

June 25th, 1900, assisted by Drs. McHatton, Ross and Derry, the writer performed a typical resection of the hip-joint. The head and great trochanter of the femur, which contained a diseased center, was removed, the track of the old sinus in the thigh and all diseased tissues in the capsule of the joint were cut away. The acetabulum was found to be intact. The wound was irrigated with sterilized salt solution and scrubbed with gauze mops. The cavity was filled with an iodoform tampon, the wound closed with silk-worm sutures, iodoform dressings employed, and Buck's extension applied to the limb. Twenty-four hours later the tampon was removed and a ten per cent. emulsion of iodoform was injected and the opening closed. Primary union occurred, notwithstanding a severe dermatitis, some delirium and slight fever (temperature 100 to 101) due to the iodoform dressings. Owing to the child's general good condition and the intensely hot weather, she was allowed to get up on crutches two weeks after the operation, the limb being encased in a wire splint, a high-soled shoe worn on the sound foot, and a lead weight (1 lb.) attached to the shoe of the diseased limb.

For two months this mechanical treatment was continued. Cod-liver oil with creosote, iodide of iron, generous dieting, and out-of-door life constituted the constitutional treatment.

September 1st, she was allowed to abandon the crutches, high shoe and weight. A good artificial joint was established, with restored health.

Two months later, while in perfect health, she was seized with diphtheria, and notwithstanding the best of treatment by her family physician, Dr. Ross, she died within forty-eight hours of its onset.

The following is the microscopical report of Dr. T. E. Oertel, of Augusta, who kindly made the sections of the diseased bones:

Irregular pieces of bony tissue, about 2 x 3 cm. in size, spongy in character, covered on one aspect with a dense, white structure, marking the outer portion of the bone. Several of the pieces exhibit a dark-colored, more or less irregular area, involving in each case the outer aspect of the bone. This tissue has the appearance of that usually found lining a pyogenic cavity or sinus.

Pieces of the bone were decalcified, cut by the celloidin method and stained in hematoxylin and eosin.

MICROSCOPICAL EXAMINATION.

In general there is seen the structure of normal spongy bone consisting of trabeculæ with interspaces filled with a delicate connective tissue framework, fat, blood and round cells, the number of the latter somewhat increased above the normal standard.

Those portions of the outer surface that were rough and dark in color are found to consist of a dense fibrous tissue filled with numerous free, irregularly shaped pigment granules and here and there small areas of new-formed connective tissue cells.

In some portions of this tissue are areas that take the nuclear stain pronouncedly and consist of aggregations of small, round lymphoid cells intermingled with epithelioid connective tissue cells.

The centers of these areas are hyaline and have evidently undergone coagulation necrosis.

These areas present the characteristics of yellow tuberculosis.

In the next case we trace the successive stages of tuberculosis in a child having an hereditary diathesis, illustrating the metastatic course of the process and the almost invariable result of early neglect. Nor does the writer believe the results obtained is the end of the tubercular invasion in the case. Sooner or later pulmonary tuberculosis or infection in other organs will develop, not as a post-operative infection, nor as an auto-infection from the diseased hip; this focus has been removed and the operation was complete. *The*

*new invasion will be from the tubercle bacilli, latent elsewhere in the boy's organism at the time of the operation, and of course still present.*

CASE 2.—C. K., male, aged five years, mulatto, of poor family history, had scars of past suppurating cervical glands and of a corneal ulcer of the left eye. The glandular trouble began when the child was twelve months of age, the corneal ulcer about six months later. When three years of age he began limping, had pain in the left knee, and later the left hip was swollen. At the age of four years there was a dislocation of the hip and adduction of the limb.

July 14th, 1900, he entered the Macon Hospital, emaciated, had hectic fever, and two suppurating sinuses of six months' duration leading to the hip-joint, one near the middle of the thigh, the other over the lower left border of the sacrum. There was no lung or other involvement discoverable.

July 17th, assisted by Dr. Carswell and Dr. Wright, a complete excision was performed by the writer, after all granulating tissues in the acetabulum, the capsule of the joint and the sinuses being first curetted. Two sequestra from the head of the femur and a portion of its cartilage were loose in the cavity of the joint. The remains of the femoral head were removed through the anatomical neck in sound bony tissue.

The dressings and after-treatment of this case were the same as the previous case, but the child was confined to bed for a much longer time. At the end of three months crutches were abandoned. The child is now apparently in good health and growing rapidly, using the newly formed hip-joint with apparent ease and comfort.

Unfortunately, the anatomical specimens of this case were lost.

These cases illustrate the fact that good fibrous ligamentous joints can be obtained even after complete excision of the head of the femur and the capsular ligaments, if the after-treatment of the cases is so directed. Shortening, nevertheless, will occur, in the last case, should the boy live to adult life, and would have resulted in the first had she lived. The bootmaker, however, can now easily remedy the discomforts of a shortened limb.

Early excision is most urgently indicated in the wrist-joint infected with tuberculosis; arthrectomy should never be attempted, as complete eradication of all foci of disease is impossible by this method. Warren claims that even resection is a useless measure in tuberculosis of the carpus and tarpus in adults, owing to the dangers of auto-infection and the usually useless member left by the operation. It is a well-known fact that caries of the wrist is more often followed by pulmonary tuberculosis than is the disease located in other joints; particularly in adults is this true, in whom these joints are most frequently involved and the outlook from any form of tuberculosis is always most hopeless. I agree with him that excision or any operation short of amputation in the ankle is discouraging, owing to the average faulty function results; still I am convinced that early radical excision of the wrist will give protection against auto-infection. If the operation is undertaken before the parts are destroyed and the fingers are not stiffened by inflammatory deposits around the tendons, a fairly useful member may be saved. In the case which follows, fortunately the disease, though far advanced, had not yet completely destroyed the joint nor entirely crippled the fingers, and a successful excision could be performed. Functionally the results are fairly good, and the writer feels fairly sure that this man's death will not be from auto-infection from his wrist, but if he dies from tuberculosis it will be the result of latent foci present elsewhere at the time of the operation.

Case 3.—P., male, aged thirty-nine years; white farmer, of good family and previous history; began in the winter of 1899 to have pain and swelling in the left wrist. A short time previous to this he had cut one of his fingers while skinning a dead cow. An indolent wound followed, but finally healed. His physician in the spring incised the back of the swollen wrist, letting out some fluid. This incision quickly closed, but the pain and swelling was unrelieved, and he lost weight and strength.

October 25, 1900, he came to the writer for treatment. The wrist was swollen and edematous, the joint being spindle-shaped. There were no fistulæ or openings communicating with the joint. Pain was constant, preventing sleep. Motion in the joint was limited, but the fingers could be fairly closed. He had no cough or other evidence of constitutional or local involvement, and his general condition was good except that he was off in weight some twenty-five pounds.

November 1, at the Macon hospital, assisted by Dr. McHatton and Dr. Carswell, the writer operated. The line of incision was made along the dorsum of the wrist and hand, beginning a little to the ulnar side of the middle of the articular end of the radius, and extending across the carpus down the hand nearly to the web between the second and third fingers. The tendons of the extensor communis digitorium were pushed to either side and the joint and hand split between the trapezoid and os-magnum, the scapoid and the semilunar and between the second and third metacarpal bones. By this means the deep parts were widely opened; care was taken not to carry the incision to the deep tissues of the palm, so as to avoid cutting the two palmar arches or to weaken the strength of the hand. In this wound the heads of two metacarpal bones and all of the carpal bones except the pisiform were removed. The articular end of the radius was not diseased and was not removed. Only the two extensor tendons to the carpus at their insertions were cut. Much tubercular débris in the joint and gelatinoid tissue about the tendons of the hand were removed. Esmarch's bandage above the elbow made the operation bloodless.

This method is Dr. Wm. J. Taylor's modification of Mynter's procedure in performing excision of the wrist-joint, and is by far the easiest and most conservative method of approaching this joint (*Annals of Surgery, September, 1900*).

The wound was washed and scrubbed out thoroughly, packed with iodoform gauze, closed with silkworm-gut sutures, except at the most dependent point for drainage, and the hand dressed and placed on a wire palmar splint. In forty-eight hours the packing was removed, iodoform emulsion injected and the drainage wound closed. The patient left the hospital on the sixth day and made an uneventful recovery under proper constitutional treatment. He now had a fairly useful hand, has recovered in weight what he had lost (twenty-five pounds), and feels perfectly well.

Dr. Oertel submits the following microscopical report:

The specimen submitted consists of a number of large fragments of carpal bones, presenting the usual characteristics of these bodies. The articular surfaces of some of the fragments are partially covered by a more or less dense and tough white tissue. This is of a fibrous nature and quite elastic.

The largest mass of this description measures 1.5x.5 cm. This is sectioned by the celloidin method and stained with hematoxylin and eosin.

MICROSCOPIC CHARACTERISTICS.

The mass consists in general of irregularly disposed bundles of wavy fibrous tissue, for the most part devoid of nuclei. Small aggregations of fat are noted and in these situations there are arteries with greatly thickened walls, in many instances the lumen of the vessels being entirely obliterated.

About these vessels are numerous small round (lymphoid) cells and new connective tissue cells, having no definite form of arrangement. The tissue described merges upon one side with bony trabeculæ in which nothing abnormal is noted. No definite tubercle formation is seen. The process is evidently inflammatory in nature.

One of the smaller pieces of carpal bone after being decalcified was sectioned by the celloidin method, stained in hematoxylin and eosin and mounted in balsam. The sections presented the appearance of the normal structure of the part.

The doctor's failure to find absolute evidence of tubercle formation is no fault of his methods, nor is it conclusive evidence of the absence of tuberculosis in the wrist. The disease was not universal, some of the bones were not involved. All of the bones were not sent to him, and only two or three specimens he received were examined by him, owing to lack of time.

---

A HOME remedy for chronic constipation in infants got the scientific sanction by Doerfier (*Munich Med. Wochenschrift*). He found that in infants over one month the addition of one-half to one teaspoonful of fresh butter given every morning and evening till a normal stool is secured will be very often sufficient. After bowel movement comes regularly and consisting of not hardened feces, the double dose should only be given every second day and further the amount gradually increased as the child grows older and the necessity requires.

## Figures and Tables

**Figure f1:**
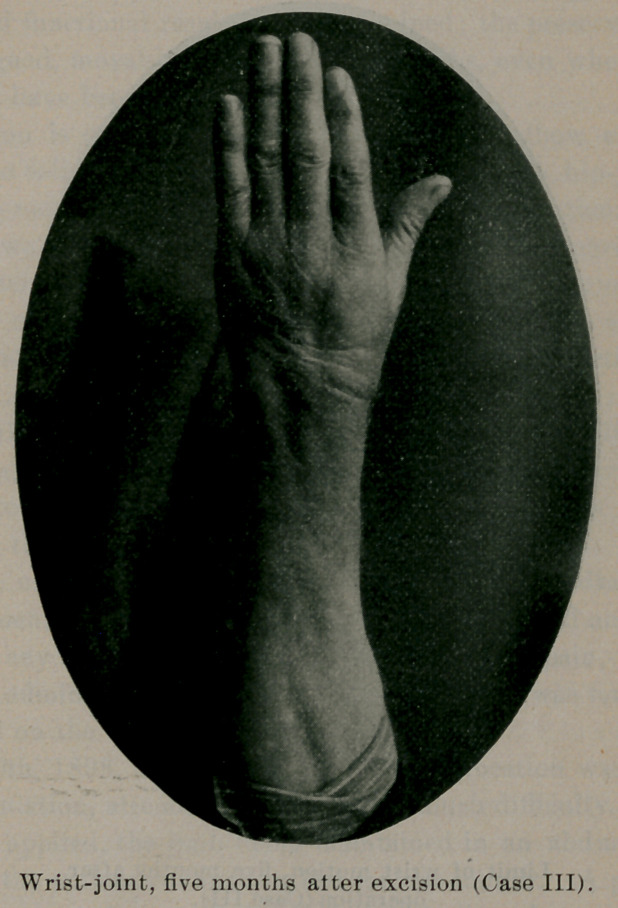


**Figure f2:**